# Coding mechanisms for diagnosis timing in the International Classification of Diseases, Version 11

**DOI:** 10.1186/s12911-022-01990-8

**Published:** 2022-09-16

**Authors:** Vijaya Sundararajan, Marie-Annick Le Pogam, Danielle A. Southern, Harold Alan Pincus, William A. Ghali

**Affiliations:** 1 Department of Medicine, St Vincent’s Hospital, University of Melbourne, Melbourne, Australia; 2 Department of Epidemiology and Health Systems, Center for Primary Care and Public Health (Unisanté), University of Lausanne, Lausanne, Switzerland; 3 Centre for Health Informatics, Cumming School of Medicine, University of Calgary, Calgary, Canada; 4 Department of Psychiatry, Vagelos College of Physicians and Surgeons, Columbia University and New York State Psychiatric Institute, New York, NY USA; 5 Department of Medicine, University of Calgary, Calgary, Canada

**Keywords:** Diagnosis timing, International Classification of Diseases, Quality and safety

## Abstract

**Background:**

Diagnoses that arise after admission are of interest because they can represent complications of health care, acute conditions arising de novo, or acute decompensation of a chronic comorbidity occurring during the hospital stay. Three countries in the world have adopted diagnosis timing codes for a number of years. Their experience demonstrates the feasibility and utility of associating an International Classification of Diseases, Version 9 or International Classification of Diseases, Version 10 diagnostic code with information on diagnosis timing, either as part of a diagnostic field or as a separate field. However, diagnosis timing is not an integrated feature of these two classifications as it will be for International Classification of Diseases, Version 11.

**Methods:**

We examine the different types of diagnosis timing that can be used to describe complex patients and present examples of how the new International Classification of Diseases, Version 11 codes may be used.

**Results:**

Extension codes are one of the important new features of International Classification of Diseases, Version 11 and allow more specificity in diagnosis timing.

**Conclusion:**

Imbedded and standardized diagnosis timing information is possible within the International Classification of Diseases, Version 11 classification system.

## Background

Coded hospital morbidity data are used internationally for many reasons, including in the assessment of quality of care and healthcare provider performance. A feature in such data, which substantially facilitates the capture of quality and safety events within health care settings, is the ability to time the onset of conditions. In particular, diagnosis timing allows users to distinguish whether a diagnosis was present on admission or arose during the hospital stay.

A patient is admitted to hospital with osteoarthritis and has an ST-elevation myocardial infarction (STEMI) during his stay. He has a history of several comorbid conditions, including type 2 diabetes (DM) and chronic obstructive pulmonary disease (COPD). During the stay, he develops diabetic ketoacidosis resulting from his STEMI.

In the example above, osteoarthritis would be coded as the reason for admission. DM and COPD would also be coded as present on admission as they require treatment and monitoring during the patient stay. The acute STEMI and the diabetic ketoacidosis would be coded as diagnoses that developed after admission.

As the example highlights, information about the timing of a condition in relation to admission is an important feature to capture along with specific conditions. Diagnoses that arise after admission are of interest because they can represent complications of health care, acute conditions arising de novo, or acute decompensation of a chronic comorbidity occurring during the hospital stay [1, 2]. An advantage of diagnosis timing is the ability to redefine patient safety indicators because diagnosis timing information allows simplification of exclusion criteria [3, 4].

Diagnosis timing is currently a facet of routinely collected health information in Canada, Australia, and the United States. Canada first introduced a mandatory diagnosis timing field, called “Diagnosis typing” in 1976 while diagnostic coding was performed using ICD-9 (Table 1) [5]. One Australian state introduced diagnosis timing capability in 1992, followed by three US states between 1994 and 2002 [5, 6]. Nationally, the “Diagnosis Onset Type” was introduced in the ICD-10-Australian Modification in 2006 [7, 8] and a “Present on Admission” flag was implemented in the United States at the end of 2007, with the adoption of ICD-10-Clinical Modification [6].

**Table 1:** Chronology of adoption: diagnosis timing indicators

Year	Location	Field	Classification	Categories
1976	Canada	Diagnosis type	ICD-9-CM and ICD-10-CA	In Canada, the indicator is a single-digit numerical code.:“M” for most responsible diagnosis/main condition;. . “Type (1)” for a condition that existed pre-admission, comorbid conditions that were active and notable during a stay; “Type 2” for a condition that has arisen after admission; “Type (3)” for a condition for which a patient may or may not have received treatment, but which is a comorbidity; and “Type (4)” for a morphology code
1992	Victoria, Australia	Vic Prefix	ICD-9-CM and ICD-10-AM	“P” for a primary diagnosis for which the patient received treatment or investigation; “A” for an associated condition that may have been the underlying disease for the condition being treated; “C” for a condition that was not present at the time of admission; and “M” for a morphology code
1994	California, USA	Condition Present on Admission Modifier	ICD-9-CM	The POA field, one for each diagnosis field, could take on one of three values: “1” for a diagnosis that was present on admission to hospital; “2” for a diagnosis not present at admission, and a state-specific value for “uncertain or unknown.”
1996	New York, USA		ICD-9-CM	
2002	Wisconsin, USA		ICD-9-CM	
2006	Australia	Diagnosis Onset Type	ICD-10-AM	“1” for primary condition; “2” for post-admit condition; and “9” for unknown or uncertain
2007	USA	Present on Admission	ICD-10-CM	“Y” for present on admission; “N” for not present on admission; “U” for insufficient information; “W” for clinically undetermined; and “1” for exempt from POA
2008	Australia	Condition Onset Flag	ICD-10-AM	“1” for condition with onset during the episode of admitted patient care; “2” for condition not noted as arising during the episode of admitted patient care; and “9” for not reported

Adapted from [5]

As the experience of these countries demonstrates, it is clearly possible to associate an ICD-9 or ICD-10 diagnostic code with information on diagnosis timing, either as part of a diagnostic field or as a separate field. However, diagnosis timing is not an integrated feature of these two classifications.

In the spirit of providing features that allow for the accurate description of healthcare, ICD-11 has taken the approach of post-coordination of diagnoses with the use of extension codes. This is in contrast to the pre-coordination approach in ICD-9 and ICD-10, in which sets of predefined codes are available to describe complex clinical conditions.

In this article, we examine the different types of diagnosis timing within the broad context of extension codes in ICD-11 that can be used to describe complex patients, such as the one in the opening example who is admitted for one condition, then experiences another condition during admission and also has concurrent illness. Further examples of how ICD-11 now provides this feature internationally are also presented.

## Main text

As noted in a prior article in this series, there are two main types of extension codes [9]: type 1 codes allow the addition of extra detail to the stem code, whereas type 2 codes are diagnosis code descriptors [10]. The stem and extension structure allows efficiency, flexibility, and detail in the way information is captured based on a limited number diagnosis codes without prespecified combinations. Although stem codes may be used in isolation, an extension code must always be combined with a stem code.

The meaning of the final ICD-11 code is based on the condition within the stem code; the use of a type 2 diagnosis code descriptor extension code provides more information regarding the condition’s importance, timing, method/certainty of its diagnosis, and context. The meaning of the code refers to the same condition, but the use of a type 2 diagnosis code descriptor extension code alters its interpretation. There are seven groups of type 2 extension codes (Table 2): Discharge diagnosis types, Diagnosis timing, Diagnosis timing in relation to surgical procedure, Diagnosis method of confirmation, Diagnosis certainty, Obstetrical diagnosis timing, and Encounter descriptions.

**Table 2:** Type 2 Extension codes: diagnosis code descriptors

Type	Code and description
Discharge diagnosis types	XY0Y Main condition: Reason for encounter or admission after study at the end of the episode
	XY7B Main resource condition
	XY6E Initial reason for encounter or admission
Diagnosis timing	XY6M Present on admission
	XY69 Developed after admission
	XY85 Uncertain timing of onset relative to admission
Diagnosis timing in relation to surgical procedure	XY9U Preoperative
	XY9N Intraoperative
	XY7V Postoperative
Diagnosis method of confirmation	XY3B Diagnosis confirmed by laboratory examination
	XY0E Diagnosis confirmed by serology
	XY9Q Diagnosis confirmed by histology
	XY8K Diagnosis confirmed by genetics
	XY9R Diagnosis confirmed by imaging
Diagnosis certainty	XY7Z Provisional diagnosis
	XY75 Differential diagnosis
Obstetrical diagnosis timing	XY3K Delivered with or without mention of antepartum condition
	XY8Q Delivered, with mention of postpartum condition
	XY8U Antepartum condition or complication
	XY9P Postpartum condition or complication
	XY9S Unspecified as to episode of care, or not applicable
Encounter descriptors	XY18 Initial encounter
	XY8S Subsequent encounter

In brief, there are three categories within the discharge diagnosis types: main condition, main resource condition, and initial reason for encounter or admission. The main condition [11] relates to the episode of hospital-based care and should be identified and recorded as the “one condition that is determined to be the reason for admission, established at the end of the episode of health care”. This determination is supported through evaluations and investigations that aim to establish the diagnosis responsible for the admission. More extensive discussion is found within a later paper in this series on main condition (Discharge diagnosis types).

Although some inherent timing information is within the main condition and main resource coding extension codes, further diagnosis timing specificity is possible in relation to admission and surgical procedures. ICD-11 will use the following three categories to capture the timing of diagnoses via an extension code [12]:

1. XY6M Present on admission,
2. XY69 Developed after admission, and
3. XY85 Uncertain timing of onset relative to admission.

The timing of diagnoses associated with a surgical procedure is also possible with the following three codes:

1. XY9U Preoperative,
2. XY9N Intraoperative, and
3. XY7V Postoperative.

Whereas diagnosis timing is core to the recommendations of ICD-11 with regard to implementation, diagnosis timing in relation to surgical procedure is supplementary information and may be reserved for later implementation in countries that want to use these extension codes but face constraints in changing their health information infrastructure.

We present some examples of post-coordination using diagnosis timing extension codes from the ICD-11 Reference Guide [12]. Of note these examples are not based on real life patients.

Example 1: A patient with long-standing type 1 diabetes is admitted to hospital because of chest pain, which upon assessment is diagnosed as a myocardial infarction. The patient develops a deep vein thrombosis in the right lower limb as an in-hospital complication of care.

In this example, both diabetes and myocardial infarction are present at admission, but the myocardial infarction does not need to be coded as being “present on admission” because it is the main condition, designated in this example as being “the condition that is determined to be the reason for admission, established at the end of the episode of health care.” For the in-hospital complication, a diagnosis timing extension code for “developed after admission” is linked by cluster coding to a stem code for “deep vein thrombosis.”

The appropriate coding (Table 3) of this scenario includes a combination of three clustered coding entities, each of which involves a stem code linked to an accompanying extension code:

- Stem code for BA41.Z (acute myocardial infarction, unspecified) & extension code for XY0Y (main condition);
- Stem code for 5A10 (diabetes mellitus type 1) & extension code for XY6M (present on admission); and
- Stem code for BD71.4 (Lower limb deep vein thrombosis) & extension code for XK9K (Right) & extension code for XY69 (developed after admission)

**Table 3:** ICD-11 coding for example 1, A patient with long-standing type 1 diabetes is admitted to hospital because of chest pain, which upon assessment is diagnosed as a myocardial infarction. The patient develops a deep vein thrombosis in the right lower limb as an in-hospital complication of care

Diagnosis1	Diagnosis2	Diagnosis3
BA41&XY0Y	5A10&XY6M	BD71.4&XK9K&XY69

Note that for all three coded entities in the above example, an ampersand (&) is used. In the first cluster, the stem code for myocardial infarction is linked to a diagnosis type extension code for main condition diagnosis type. In the second cluster, the stem code for diabetes mellitus type 1 is linked to a diagnosis timing extension code for present on admission. In the third cluster, the stem code for lower limb deep vein thrombosis is linked to extension codes for right side of the body and one for developed after admission.

Example 2: A patient with long-standing type 2 diabetes is admitted to hospital after developing hypoglycaemia, which is noted to be a result of liraglutide by the medical team. On day 3 during her hospital stay, the patient has a fall out of the hospital bed, with a fracture to her right hip.

The main condition is hypoglycaemia. Other conditions include diabetes mellitus, type 2; a fall after admission; and a right femoral neck fracture during the hospital stay. For the in-hospital fall in this example, a diagnosis timing extension code for “developed after admission” is linked by post-coordination to a stem code for “fall” and to a stem code for “hip fracture” (Table 4). Moreover, the three-part model for coding quality and safety events [13] including: (1) a healthcare-related activity that is the ‘source or context’ of harm; (2) a ‘mode’ or ‘mechanism’ of harm; and (3) a harmful consequence of the event (most importantly injury or other harm to the patient) is also used:

- Stem code for 5A21.0 (Hypoglycaemia in the context of diabetes mellitus without coma);
- Stem code for 5A11 (Type 2 diabetes mellitus) & mandatory postcoordination extension code for XY0Y (Main condition);
- An external cause stem code for the cause, PL00 (Drugs, medicaments or biological substances associated with injury or harm in therapeutic use);
- An external cause stem code for the mode/mechanism PL13.Z (Mode of injury or harm associated with exposure to a drug, medicament or biological substance, unspecified) & extension code for XM0EQ7 (Liraglutide);
- The three-part model, with extra detail:
  - Stem code for NC72.2Z (Fracture of neck of femur, unspecified) & extension code for XY69 (Developed after admission) & extension code for activity when injured XE245 (Being taken care of by health care professional) & extension code for place of occurrence XE28K (Hospital);
  - Stem code for cause of injury PL10 (Other health care related causes of injury or harm). This is the cause in the three-part coding model of injury, cause, mode;
  - Stem code for PL14.E (Fall in health care). This is the mode/mechanism in the three-part model; and
  - Stem code for PA60 (Unintentional fall on the same level or from less than 1 m), which is an extra detail related to the fall & extension code for XE8PK (Bed).

**Table 4:** ICD-11 coding for example 2: A patient with long-standing type 2 diabetes is admitted to hospital after developing hypoglycaemia, which is noted to be a result of liraglutide by the medical team

Diagnosis1	Diagnosis2	External cause: cause and mode/mechanism	
		Cause	Mode/mechanism
5A21.0	5A11&XY0Y	PL00	PL13.Z&XM0EQ7
5A21.0/5A11&XY0Y/PL00/PL13.Z&XM0EQ7			

Quality and safety three-part model: Harm, cause, and mode/mechanism			
Harm (Diagnosis3)	Cause	Mode/mechanism	Extra detail
NC72.2Z&XY69&XE245&XE28K	PL10	PL14.E	PA60&XE8PK
NC72.2Z&XY69&XE245&XE28K/PL10/PL14.E/PA60&XE8PK			

On day 3 during her hospital stay, the patient has a fall out of the hospital bed, with a fracture to her right hip. The main condition is hypoglycaemia. Other conditions include diabetes mellitus, type 2; two falls, one before admission and one after admission; and a right femoral neck fracture during the hospital stay

Note that diagnosis timing codes (here XY69) are applied to diagnosis codes only and not to the external cause code. Hence a complex clinical presentation and hospital course can be captured.

Example 3: A patient aged 75 years old with asymptomatic bilateral carotid artery stenosis, essential systolic and diastolic hypertension, and obesity is admitted to hospital for a planned arthroplasty of the right knee as a treatment for primary osteoarthritis. This patient has been treated with low-dose aspirin for carotid atherosclerosis and put on direct oral anticoagulant after surgery to prevent venous thromboembolism. During recovery from surgery, the patient experiences a left hemisphere ischemic stroke as a postoperative complication.

In this case, the main condition is primary osteoarthritis of the right knee. Other conditions include hypertension and obesity, which are present on admission and require evaluation and treatment during hospital stay. Finally, a left hemisphere ischemic stroke occurs after surgery. A diagnosis timing extension code for “postoperative” is linked by post-coordination to the stem code for “stroke” while a diagnosis timing extension code for “present on admission” is linked by post-coordination to the stem codes for “hypertension” and “obesity” (Table 5). Note that the history of asymptomatic bilateral carotid artery stenosis should not be coded as the patient experienced a complication of atherosclerosis after surgery.

- Stem code for FA01.0 (Primary osteoarthritis of knee) & extension code for laterality XK9K (Right) & mandatory post-coordination extension code for XY0Y (Main condition)
- Stem code for 5B81.01 (Obesity in adults) & extension code for XY6M (Present on admission);
- Stem code for BA00.0 (Combined diastolic and systolic hypertension) & extension code for XY6M (Present on admission);
- Stem code for 8B11.0 (Cerebral ischaemic stroke due to extracranial large artery atherosclerosis) & extension code for laterality XK8G (Left) & extension code for XY7V (developed after surgery = postoperative).

**Table 5:** ICD-11 coding for example 3, A patient aged 75 years old with asymptomatic bilateral carotid artery stenosis, essential systolic and diastolic hypertension, and obesity is admitted to hospital for a planned arthroplasty of the right knee as a treatment for primary osteoarthritis

Diagnosis1	Diagnosis2	Diagnosis3	Diagnosis4
FA01.0&XK9K&XY0Y	5B81.01&XY6M	BA00.0&XY6M	8B11.0&XK8G&XY7V

This patient has been treated with low-dose aspirin for carotid atherosclerosis and put on direct oral anticoagulant after surgery in order to prevent venous thromboembolism. During recovery from surgery, the patient experiences a left hemisphere ischemic stroke as a postoperative complication

Note that the stem code for BD55 (Asymptomatic stenosis of intracranial or extracranial artery) is not coded here as it includes “Stenosis of intracranial or extracranial artery that has not caused TIA or cerebral ischemic stroke.”

As demonstrated in the examples above, and in accompanying articles in this series, the post-coordination approach in ICD-11 allows considerable flexibility and detail in the coding of healthcare diagnoses and events through the use of extension codes. Example 3 particularly highlights how useful the diagnosis timing extension codes of “XY6M Present on admission,” “XY69 Developed after admission,” and “XY85 Uncertain timing of onset relative to admission” can be.

Diagnosis timing and surgical timing extension codes have the ability to improve routinely collected and coded hospital discharge data to support research and the development of performance, quality, and safety indicators. The standardized approach of classifying diagnosis timing in ICD-11 will enhance the international comparability of these data in the future. A more sophisticated approach to this issue would be “time- and date-stamping” of individual diagnoses; however, such an approach can only be undertaken in health information systems that have the coding and resources to support uptake.

The ICD-11 Reference Guide very clearly lays out the history of ICD-11 and the multiple use cases for the classification and coding [14]. These include the case-mix use case for health care financing, the mortality use case (the original use case that motivated the development of coding of causes of death on a global scale), the morbidity use case (including disease surveillance), the quality and safety use case, and some others as well. Diagnosis timing features in ICD-11 will refine health information for each of the use cases, and permit better diagnostic distinctions to be made (e.g. confining hospital reimbursement systems only to pay for diagnoses present only at admission; detecting quality and safety events that arose only after the admission; or detecting only postoperative events).

There are several challenges to the introduction of diagnosis timing extension codes and the adoption of the clustering mechanism. Timing a diagnosis depends on the quality of medical record documentation and the judgment of the coder. Having options such as code XY85 for “Uncertain timing of onset relative to admission” allows coders needed choice in situations where the clinical notes do not provide enough detail with which to time a diagnosis. Additionally, as one of the primary uses for coded hospital abstract data is to develop funding models, coders may focus their efforts on diagnoses and timing that are likely to increase hospital payment. Ideally, however, coding guidelines should support complete coding of the hospital stay. Certainly, this increased completeness of coding needs to be balanced against the resources available to support this effort as such coding detail may take longer than is currently required to code a hospital record. It is possible, however, that the flexibility of post-coordination can mitigate the time required to code detailed hospital stays. Finally, the highly standardized structure of the ICD-11 may also lend itself to the implementation of automated coding from electronic health records.

Diagnosis timing, in addition to other related extension code sets, such as “Diagnosis timing in relation to surgical procedure” and “Obstetrical diagnosis timing,” have great potential to improve the quality of coding, particularly in capturing quality and safety events. The framework of post-coordination with its stem codes, its extension codes, and the use of a joiner such as an ampersand is a true innovation of ICD-11. Extension codes will allow more accuracy not only with diagnosis timing but also in distinguishing what represents a “main condition.” How well this potential can be realized in day-to-day coding is currently being investigated. Early reports suggest that while the added detail possible with extension codes may appear to place more of a burden on coding and coders, it may be balanced by the decrease in uncertainty from not being able to accurately capture the context of a diagnosis, which was less possible with the pre-coordinated codes within ICD-10.

## Conclusion

Three countries in the world have adopted diagnosis timing codes for a number of years: Canada for over 30 years, Australia for approximately 20 years, and the US more recently. The mechanisms in the three countries for diagnosis timing differed in ICD-10, and they required a special data field in their disparate information technology systems. Now ICD-11 presents a diagnosis timing feature that can be adopted internationally. Although extension codes are an optional feature for country implementations, they are certainly one of the exciting and powerful new features of ICD-11, and many countries are likely to adopt them. Accordingly, these embedded diagnosis timing options can now be used by all countries who want to use them in a standardized manner where the information is within the classification system rather than a supplementary data element.

## Abbreviations

COPD: Chronic obstructive pulmonary disease
DM: Diabetes mellitus, or type 2 diabetes
ICD: International Classification of Diseases
ICD-9: International Classification of Diseases, 9th revision
ICD-10: International Classification of Diseases, 10th revision
ICD-11: International Classification of Diseases, 11th revision
ICD-10-CA: International Classification of Diseases, 10th revision, enhanced Canadian version
ICD-10-CM: International Classification of Diseases, 10th revision, Clinical Modification (US)
STEMI: ST-elevation myocardial infarction
POA: Present on admission
